# Effects of ecological restoration measures on the distribution of *Dicranopteris dichotoma* at the microscale in the red soil hilly region of China

**DOI:** 10.1371/journal.pone.0204743

**Published:** 2018-10-24

**Authors:** Zhiqiang Chen, Zhibiao Chen

**Affiliations:** 1 State Key Laboratory for Subtropical Mountain Ecology of the Ministry of Science and Technology and Fujian Province, Fujian Normal University, Fuzhou, Fujian, China; 2 Institute of Geography, Fujian Normal University, Fuzhou, Fujian, China; 3 School of Geographical Sciences, Fujian Normal University, Fuzhou, Fujian, China; Fred Hutchinson Cancer Research Center, UNITED STATES

## Abstract

Little is known about the evaluation of ecological restoration measures using species distribution models (SDMs) at the microscale. This study investigated the effect of arbor–bush–herb mixed plantation (ABHMP) on the potential distribution of *D*. *dichotoma* using SDMs in the typical microtopographies of the red soil hilly region of China. We examined *D*. *dichotoma* growth, microtopography, and environment-related factors at the microscale. The percentages of microtopographies and *D*. *dichotoma* physiology factors increased in the order from the valley to the ridge in the *D*. *dichotoma* patches. The valley had milder temperatures, higher humidity, and more fertile soil than the ridge in the gullies. Microclimate factors were the most critical environmental factors affecting the distribution of *D*. *dichotoma*, followed by soil factors, whereas the microtopography factors had only a marginal effect. The predicted potential distribution of *D*. *dichotoma* under the ABHMP scenario was nearly 3-fold higher than the current distribution, and the suitable area was located mostly in the level trenches and the valley. ABHMP had a strong effect on the potential distribution of *D*. *dichotoma*, and SDMs proved to be a valuable tool for assessing ecological restoration measures at the microscale.

## Introduction

Plant restoration is frequently used as the primary indicator in ecological restoration programs [1]. For example, 20% vegetation cover appears to be the threshold between natural recovery and artificial restoration, when vegetation cover drops below 20%, artificial restoration is needed in China’s Fujian province [2]. Successful management of ecological restoration projects depends largely on our ability to identify the environmental factors that affect the spread of plants and to predict their potential distribution [3]. Species distribution models (SDMs), also known as ‘bioclimatic envelope models’, rely on the niche concept [4]. SDMs statistically relate the distribution of a given species to environment-related factors which typically include available resources, limiting factors and disturbances, improving the understanding of the environmental factors affecting the species, and prediction of the potential distribution of the species [5]. A wide range of algorithms have been applied in SDMs including the Generalized Linear Model, Maximum Entropy, Artificial Neural Network, Support Vector Machine, Classification and Regression trees, Random Forest, and Generalized Boosted Regression [6]. The combined use of geographic information systems (GIS) and SDMs has proven to be an effective methodology for analyzing global patterns and ecological requirements of species [7]. SDMs have potential for multiple applications in land science, ecological restoration, and ecology [5, 8]. They are often used in regional to continental scales, and frequently rely on relatively coarse-precision data sets [9]. However, detailed knowledge of the environmental factors affecting plants and their potential distribution is fundamental for conservation planning and forecasting [10]. Thus, the use of SDMs at a relatively small scale offers improved perspectives for local conservation and management activities [11].

Many regions are flat from a large-scale perspective. At a relatively small scale, however, the microtopography is often heterogeneous [12]. Microtopography, broadly defined as topographic variability at the scale of individual plants, describes surface variation within an elevation range from roughly several centimeters to several meters [13]. Microtopography can influence factors such as plant distribution [14], seedling establishment [15], soil nutrient pools and fluxes [16], and temperature and humidity [17]. Therefore, microtopography and geomorphological processes can influence microhabitat diversity [18, 19], and the manipulation of microtopography to promote plant community and ecosystem development has implications for ecological restoration measures [13]. However, to our knowledge, relatively few studies have identified environmental factors affecting plants and predicted the potential distribution of plants using SDMs to evaluate ecological restoration measures at the microscale.

The red soil hilly region of China lies between 32° N and 18° N, spanning an area of 1.13 million km^2^. The term “red soil” refers to well-drained red loams containing argillic, oxic, or plinthitic horizons and high content of Fe and Al [20]. Demographic and economic growth has put considerable pressure on the environment and resources, and many ecosystems have been overexploited and damaged in the red soil hilly region of China. A number of ecological restoration measures have been adopted to reduce these pressures [21], and *D*. *dichotoma* dominated surface plant communities some years later regardless of the ecological restoration measures applied [22]. *D*. *dichotoma*, a perennial fern of the family Gleicheniaceae, is one of the most widely distributed ferns throughout the tropical and temperate regions. *D*. *dichotoma* can resist, tolerate, or thrive in very poor soils, making it a pioneer species for ecological restoration in the red soil hilly region of China [22].

To our knowledge, this is the first study to identify the main environmental factors affecting the distribution of *D*. *dichotoma* and predict its potential distribution under an ecological restoration scenario so as to evaluate ecological restoration measures at the microscale, using SDMs. The principal objectives were to (1) obtain information about the distribution and physiological factors of *D*. *dichotoma* and environmental factors in microtopographies; (2) identify the main environmental factors affecting the distribution of *D*. *dichotoma*; and (3) predict the potential distribution of *D*. *dichotoma* under the ABHMP scenario at the microscale and assess the effectiveness of ABHMP.

## Materials and methods

### Study area

In China, the most serious soil and water loss with the longest history occurs in the red soil hilly region in Changting County, Fujian Province [20]. Based on the information provided by the Soil and Water Conservation Bureau in Changting County, ABHMP represents the chief ecological restoration measure applied in the high-to-violent soil and water loss regions in Changting County, and involves planting trees, bushy plants, and herbs such as *Schima superba*, *Liquidambar formosana*, *Lespedeza bicolor*, and *Paspalum wettsteinii* in level trenches (400 cm × 50 cm × 40 cm, 600 hm^-2^), with Ca, Mg, and P compound fertilizer applications.

**Fig 1:**
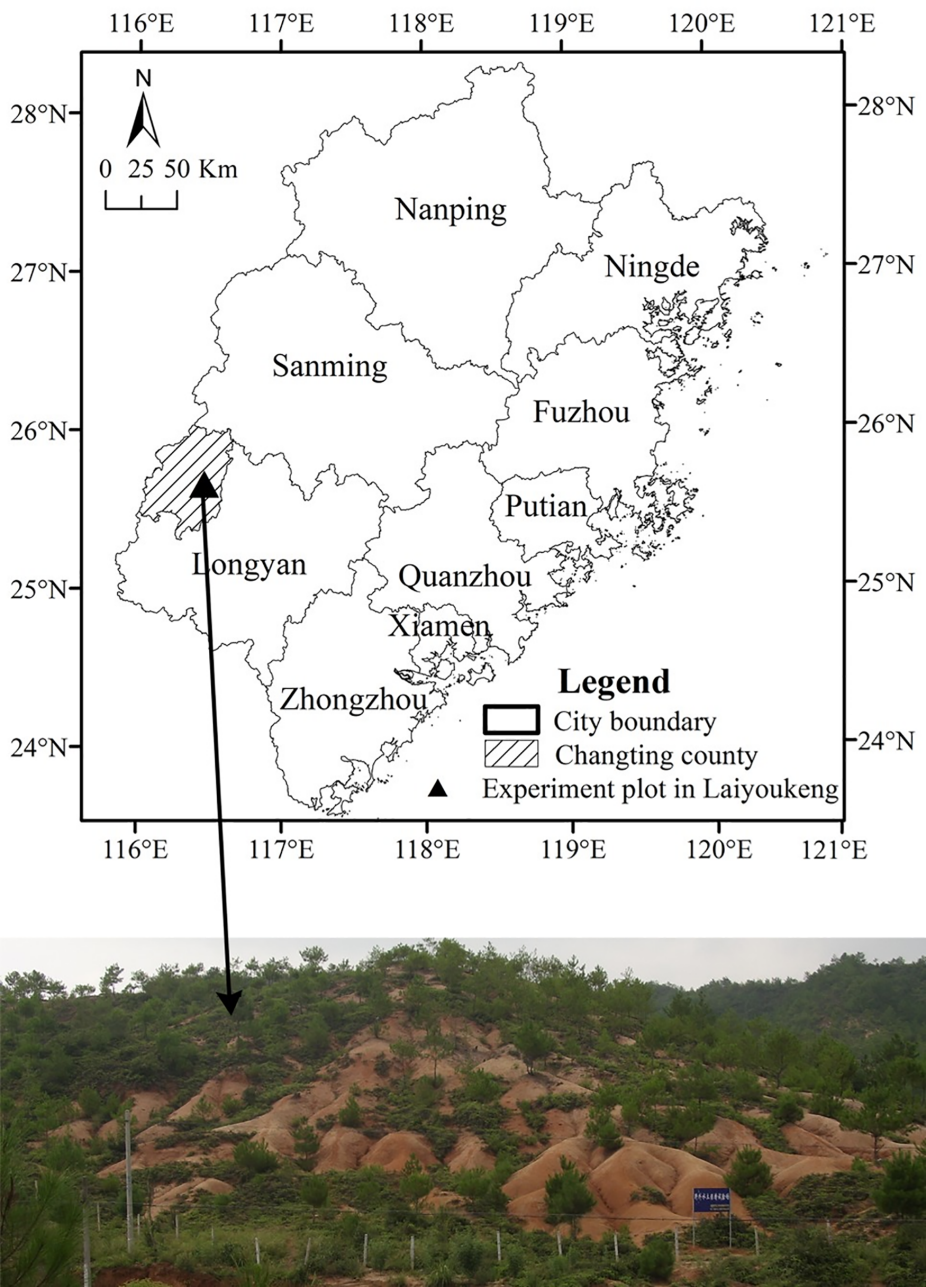
Location and photograph of Laiyoukeng.

The experimental plot in Laiyoukeng, with four stands with ABHMP in Changting County, was selected as the study area (116°23′30″ to 116°30′30″E, 25°38′15″ to 25°42′55″N). The climate of the area is subtropical and monsoon-influenced with warm and humid characteristics [20], and the soil is classified as an Alumic Ferralsol (World Reference Base for Soil Resources). The area of Laiyoukeng is 402.72 m^2^, the elevation ranges from 345 m to 365 m AMSL, the plant community is dominated by *D*. *dichotoma* with scattered shrubs, and the ecosystem is severely degraded (Fig 1). The four stands with ABHMP, that is, Duimountian, Longjing, Youfang, and Bashilihe were established in 2011, 2006, 2000, and 1983, respectively.

No specific permissions were required for the experimental plot in Laiyoukeng or the four stands, which are considered wasteland, and our field studies did not involve endangered or protected species.

### Methods

#### Measurement of microtopography and *D*. *dichotoma* patches

**Fig 2:**
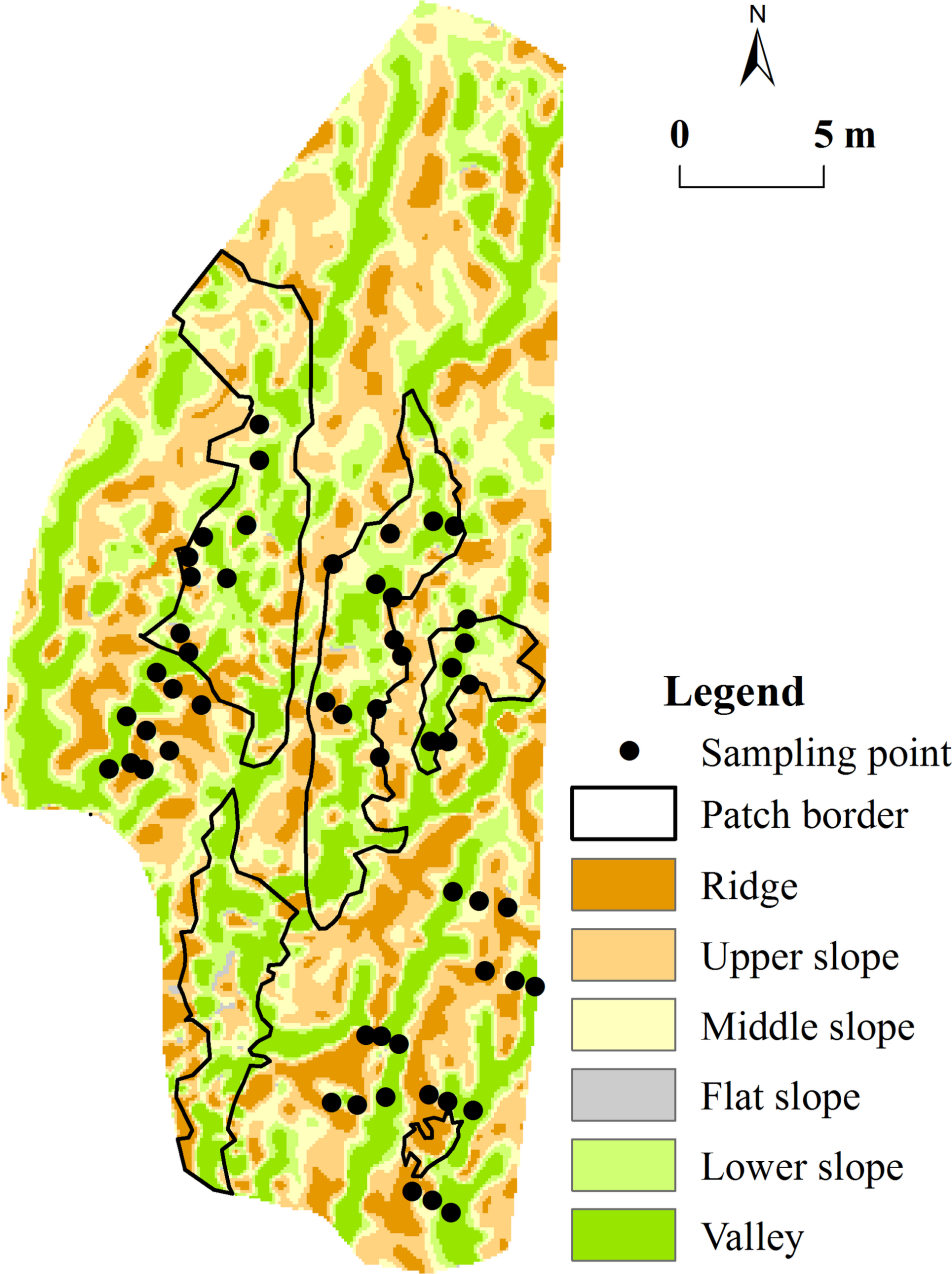
Microtopographies and sampling points in Laiyoukeng.

The locations (longitude, latitude and altitude) were measured using a Trimble 5800 GPS (mean position accuracy = ± 0.1 m) in Laiyoukeng in August, 2012 (S1 File). The number of points measured was 3,358. A point layer was created by importing a measured point, and a triangulated irregular network (TIN) layer was created from the point layer in ArcGIS10. The TIN layer was then converted to a GRID layer to produce a high-resolution digital elevation model (DEM) with a cell size of 0.1 m × 0.1 m. The Topographic Position Index (TPI), plus the slope of the cell, can be used to classify the cell into a microtopography. The TPI is the difference between a cell elevation value and the average elevation of the neighborhood around that cell. Positive values mean the cell is higher than its surroundings while negative values mean it is lower. If it is significantly higher than the surrounding neighborhood, then it is likely to be at, or near, the top of a hill or ridge. Otherwise, significantly low values suggest the cell is at, or near, the bottom of a valley. TPI values near zero may mean either a flat area or a middle slope area, and thus the cell slope can be used to distinguish the two. A method to define threshold TPI values is to use standard deviations from the elevation, which takes into account the variability of elevation values within that neighborhood. Based on both the fieldwork and our own assessment of microtopography accuracy, we used a circular neighborhood with a 1 m radius, meaning that the TPI value for each cell reflected the difference between the elevation of that cell and the average elevation of all cells within 1 m of that cell [23]. We used both TPI and the slope to form a microtopography layer that included valley, lower slope, flat slope, middle slope, upper slope, and ridge [23] (Table 1, Fig 2). The borders of the *D*. *dichotoma* patches were measured using the same method, then converted to shapefile to produce a *D*. *dichotoma* patch layer in the ArcGIS10 (S1 File). The *D*. *dichotoma* patch layer and the microtopography layer were overlain to calculate the areas and percentages of the different microtopographies in the *D*. *dichotoma* patches (Fig 2). To determine the stabilization of *D*. *dichotoma* patches, we measured the borders of the patches using a Trimble 5800 GPS once a year in August from 2012 to 2016.

**Table 1:** Classification criteria of microtopography in Laiyoukeng.

Microtopography	Classification criteria
Ridge	TPI > 0.4 SD
Upper slope	TPI > 0.15 SD and < = 0.4 SD
Middle slope	TPI > -0.05 SD and = 0.15 SD Slope > 7 degrees
Flat slope	TPI > -0.05 SD and < = 0.15 SD Slope < = 7 degrees
Lower slope	TPI > -0.3 SD and < = -0.05 SD
Valley	TPI < = -0.3 SD

Note: SD, standard deviation from elevation.

#### Location of sampling points

Three gullies with, and three gullies without, *D*. *dichotoma* were chosen for sampling, and three types of microtopographies (ridge, slope, and valley) were set in Laiyoukeng. The slope included the lower slope, flat slope, middle slope, and upper slope, which were narrow and limited for sampling. Thus, 54 sampling points (nine on the ridges, nine on the slopes, and nine on the valleys in the gullies with or without *D*. *dichotoma*) were determined to distinguish the *D*. *dichotoma* physiological, soil, and microclimate factors (Fig 2).

#### Environment factors

Three microtopography factor layers were derived from the DEM layer: altitude, slope, and aspect layers (S1 Table).

A metal ring (diameter 35 cm) was placed at each sampling point for *D*. *dichotoma* physiological and soil factors in August 2012. According to our previous study [24], we selected the mean plant height (PH), aboveground biomass per unit area (ABPUA), underground biomass per unit area (UBPUA), and total biomass per unit area (TBPUA) as the *D*. *dichotoma* physiological factors. We measured the mean PH within each ring. Given that *D*. *dichotoma* is an herbaceous plant, our analysis considered only measurements relative to the rooting zone (upper = 20 cm of the soil profile). We harvested aboveground and underground plant biomass separately by digging each ring to a depth of 20 cm, and then drying and weighing the plant material to determine ABPUA, UBPUA, and TBPUA (S1 Table).

Soil was sampled and pooled into a composite sample from the base of *D*. *dichotoma* to a depth of 20 cm within each ring. A set of eight soil factors, including organic matter, total N, available N, total P, available P, total K, available K, and pH were selected and measured. We determined organic matter by the method of oxidation with potassium dichromate in a heated oil bath; total N by means of alkali distillation; total P by means of atomic absorption spectrophotometry; total K by digestion with hydrofluoric acid and perchloric acid; available N, available P, and available K by the alkaline KMnO_4_ method, Bray’s P I method, and NH_4_OAc method, respectively; and we measured pH with a pH meter at a soil/water ratio of 1:2.5. All the soil analyses were carried out according to standard guidelines [25] (S1 Table).

*D*. *dichotoma* germinates in spring, grows in the summer and autumn, and withers in winter [26]. Based on expert knowledge from the Soil and Water Conservation Bureau of Changting County, we distinguished the following three periods: spring (March–May), summer and autumn (June–November), and winter (December–February of the following year). As plants and soil had been removed and destroyed in the sampling points for the *D*. *dichotoma* physiological and soil factors in August 2012, each sampling point for the microclimate factors was located approximately 0.1 m from the sampling point for the physiological factors. Two kinds of sampling points were located in the same microtopography to minimize differences. We measured the underground (5 cm) temperature (UT) and moisture (UM) using a soil hygrothermograph (RR-7210, mean temperature accuracy = ± 0.2°C, mean humidity accuracy = ± 3%) at the sampling points for the microclimate factors in the spring (4/22-4/29), summer and autumn (6/29-7/6), and winter (1/25-2/1) of 2015, using a 10-min time interval (S1 Table).

We sampled and analyzed the soil and microclimate factors using the same methods in the level trenches in Duimountian.

#### Species distribution models

The *D*. *dichotoma* patch layers were converted to the point layers using ArcGIS10 and 13,639 points for presence and 44,707 points for absence of *D*. *dichotoma* were generated.

Three types of environmental factors were collected. (1) Microtopography factor layers: altitude, slope, and aspect. Slope layer and aspect layer were generated based on DEM using the Slope and Aspect functions in ArcGIS10. The aspect layer was reclassified and valued as sunny slope (135–225°)-4, half-sunny slope (45–135°)-3, half-shady slope (225–315°)-2, and shady slope (0–45°, 315–360°)-1; (2) soil factor layers: raster layers for 8 soil factors were created by the Geostatistical Analyst method in ArcGIS10 using 54 sampling points; (3) microclimate factor layers: the mean, maximum, and minimum UT and UM values were calculated for spring, summer and autumn, and winter to generate 18 microclimate factors whose raster layers were created by the Geostatistical Analyst method in ArcGIS10 using 54 microclimate sampling points. Ordinary Kriging interpolation treatments were applied to eight soil factors and 18 microclimate factors by the Geostatistical Analyst method in ArcGIS10. The mean prediction errors and root-mean-square standardized prediction errors were used to validate the accuracy of raster layers generated by the ordinary Kriging interpolation, and the result was satisfactory. The raster layers are not shown due to limited space (S2 File).

**Fig 3:**
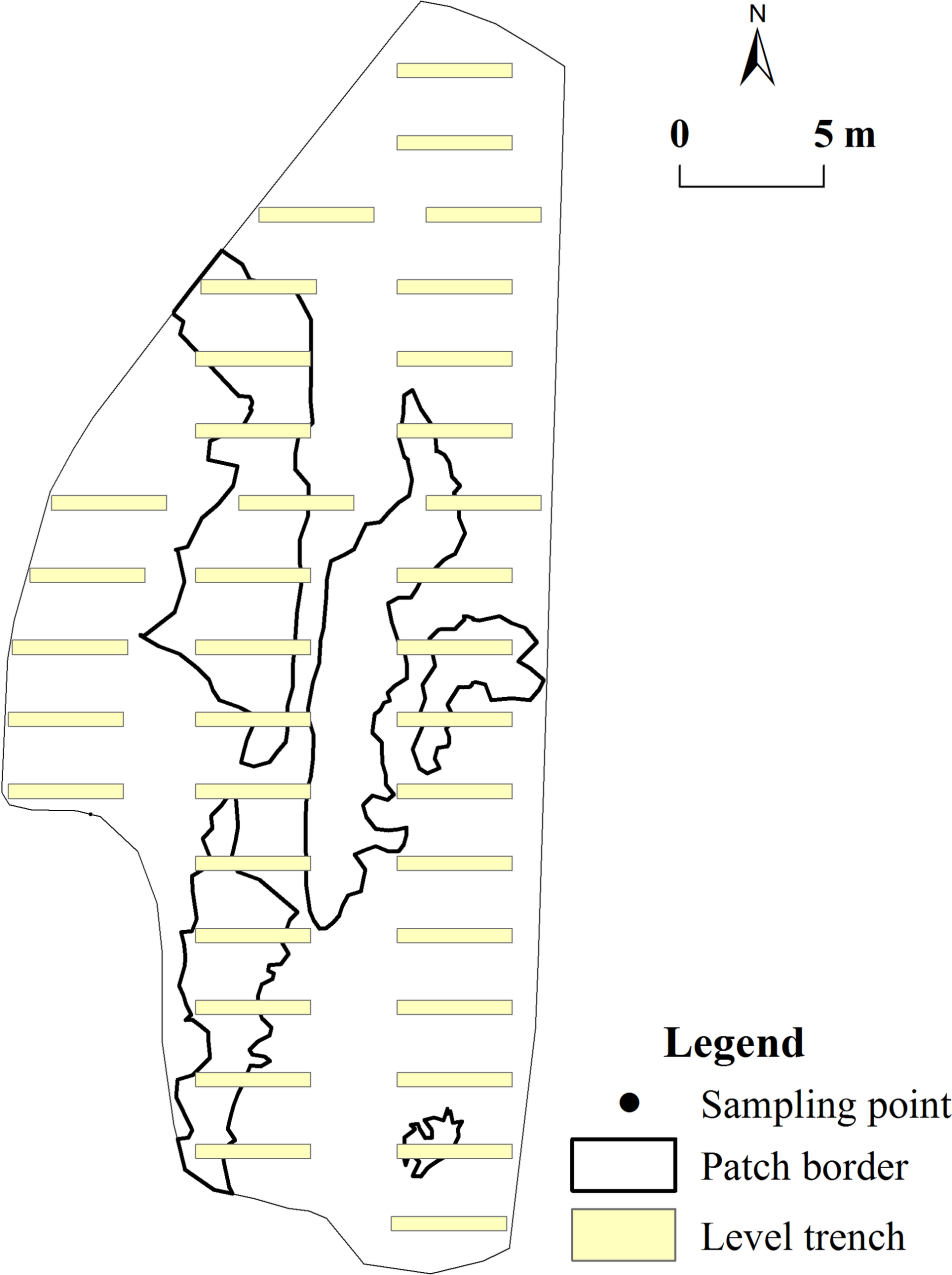
ABHMP scenario in Laiyoukeng.

We designed the ABHMP scenario and generated the level trench layer in Laiyoukeng (Fig 3 and S1 File). Based on expert knowledge from the Soil and Water Conservation Bureau of Changting County, the eight soil factors and 18 microclimate factors of the level trenches were valued according to the average values of the corresponding factors from the level trenches in Duimountian. We overlaid the level trench layers and the raster layers for three microtopography factors, 8 soil factors, and 18 microclimate factors to generate the new raster layers in Laiyoukeng. Accordingly, we obtained the new environmental factor layers under the ABHMP scenario to predict the potential distribution of *D*. *dichotoma*.

All models were run using the ModEco Platform. ModEco is a software package for species distribution modeling which provides a user-friendly platform enabling users to model species distribution data with relative ease, and includes relatively comprehensive tools for data visualization, feature selection, and accuracy assessment [27]. The four presence and absence models for species used were as follows: Generalized Linear Model, Maximum Entropy, Artificial Neural Network, and Support Vector Machine. We first calibrated the four models with current environmental factor layers and then ran the four models using the new environmental factor layers to generate the probability layers of *D*. *dichotoma* under the ABHMP scenario. Some researchers have advocated the use of combinations of multiple models. However, it has been concluded that when a single best model can be identified, incorporation of other models will bias the final model away from the best model’s predictions [28]. Therefore, we did not use combinations of models.

Model results were evaluated using the area under the curve (AUC), which is a nonparametric threshold-independent measure of accuracy commonly used to evaluate SDMs [29]. An approximate guide for classifying the accuracy of models using AUC is: excellent AUC > 0.9, good 0.9 > AUC > 0.8, fair 0.8 > AUC > 0.7, poor 0.7 > AUC > 0.6 and fail 0.6 > AUC > 0.5 [30]. We selected the models with AUC > 0.9 that have a strong predictive performance among the four models. The ABHMP scenario in Laiyoukeng was the same as the ABHMP used in the four stands (Duimountian, Longjing, Youfang, and Bashilihe), which to some extent represented the spread of *D*. *dichotoma* in the future. Thus, we compared the potential distribution of *D*. *dichotoma* under the ABHMP scenario in Laiyoukeng with that of Duimountian, Longjing, Youfang, and Bashilihe by visual examination to select the best model and its probability layer according to the similarity of the distribution of *D*. *dichotoma* in microtopographies. We reclassified the selected probability layer into the classification layer that included three types: ‘Suitable area’, ‘Sub-suitable area’, and ‘Unsuitable area’ for *D*. *dichotoma*.

We evaluated the importance of the factor values for the distribution of *D*. *dichotoma* using the selected best model and the current environmental factor layers.

#### Plant quadrat

Three representative standard plots (20 m × 20 m) were established, and four subplots (1 m × 1 m) were placed along a diagonal line in each plot in Duimountian, Longjing, Youfang, and Bashilihe. We visually estimated the vegetation cover (VC) of *D*. *dichotoma* in each subplot and the averages were obtained from the VC of *D*. *dichotoma* in Duimountian, Longjing, Youfang, and Bashilihe.

#### Statistical analyses

Normality and homogeneity were verified using Kolmogorov-Smirnov’s test and Levene’s test, respectively, prior to analysis. When necessary, data were natural log-transformed to meet the assumption of normality and homogeneity [31]. A one-way analysis of variance (ANOVA) with least square difference (LSD) was used to compare differences among *D*. *dichotoma* physiological factors, soil factors, and microclimate factors. Significance levels were set at *P* = 0.05. All statistical analyses were performed using SPSS software.

## Results

### The distribution and physiological factors of *D*. *dichotoma* and environmental factors in microtopographies

The *D*. *dichotoma* patches were very stable from 2012 to 2016, covering an area of 138.82 m^2^, equivalent to 30.09% of the total area of Laiyoukeng. The percentages of microtopographies decreased in the order from the valley through the slope (the upper slope, the middle slope, and the lower slope) to the ridge in the *D*. *dichotoma* patches (*P* < 0.05). We did not analyze the flat slope due to its negligible percentage (Table 2).

**Table 2:** Areas and percentages of microtopographies in the *D*. *dichotoma* patches in Laiyoukeng.

Areas and percentages	Valley	Lower slope	Flat slope	Middle slope	Upper slope	Ridge
Area m^2^	40.04	33.44	0.77	27.97	24.74	11.86
Percentage %	29.64 ± 3.84a	23.45 ± 1.02ab	0.61 ± 0.46c	20.08 ± 2.57b	17.09 ± 1.19bc	9.14 ± 1.09c

Different letters in the Percentage column indicate significant differences at *P*<0.05.

The four *D*. *dichotoma* physiological factors (PH, ABPUA, UBPUA, and TBPUA) decreased in the order from the valley through the slope to the ridge in Laiyoukeng (*P* < 0.05) (Table 3).

**Table 3:** Means (±SE) of *D*. *dichotoma* physiological factors among microtopographies in Laiyoukeng.

Physiological factors	Microtopography		
	Valley	Slope	Ridge
PH cm	43.38 ± 3.84a	25.44 ± 2.26b	11.64 ± 1.12c
ABPUA g·m^−2^	1054.63 ± 155.62a	434.35 ± 61.55b	110.28 ± 18.50c
UBPUA g·m^−2^	355.24 ± 125.84	250.38 ± 63.38	88.80 ± 9.19
TBPUA g·m^−2^	1409.86 ± 260.67a	684.72 ± 51.80b	199.08 ± 20.58c

Different letters in the same column indicate significant differences at *P*<0.05.

Among 26 soil and microclimate factors, 15 showed significant differences among the three microtopographies (ridge, slope, and valley) in the gullies with or without *D*. *dichotoma* (*P* < 0.05), and the valley was more humid and milder compared to the ridge in Laiyoukeng. The valley was more fertile in the gullies with *D*. *dichotoma*, while soil factors were not significantly different among the three microtopographies (ridge, slope, and valley) in the gullies without *D*. *dichotoma* (except for total N) in Laiyoukeng (Table 4).

**Table 4:** Means (±SE) of soil factors and microclimate factors among microtopographies in Laiyoukeng.

Factors	Gullies with *D*. *dichotoma*			Gullies without *D*. *dichotoma*		
	Valley	Slope	Ridge	Valley	Slope	Ridge
Maximum UT in spring °C	26.98 ± 0.80b	31.23 ± 0.24a	32.05 ± 1.15a	30.13 ± 0.85b	31.46 ± 0.57a	32.73 ± 0.62a
Minimum UT in spring °C	16.38 ± 0.13a	16.08 ± 0.10abc	15.88 ± 0.15bc	16.12 ± 0.08ab	15.77 ± 0.10bc	15.58 ± 0.09c
Average UT in spring °C	20.42 ± 0.15c	21.35 ± 0.11b	21.97 ± 0.08a	20.90 ± 0.09b	21.33 ± 0.08b	22.13 ± 0.10a
Maximum UT in summer and autumn °C	34.20 ± 1.30b	38.22 ± 1.29ab	40.26 ± 0.81a	36.97 ± 0.95ab	38.96 ± 0.39a	40.64 ± 0.15a
Minimum UT in summer and autumn °C	23.31 ± 0.15	23.13 ± 0.18	23.02 ± 0.13	23.07 ± 0.09	23.11 ± 0.18	23.17 ± 0.07
Average UT in summer and autumn °C	26.99 ± 0.15d	27.38 ± 0.09c	28.87 ± 0.07a	27.57 ± 0.05c	28.31 ± 0.05b	29.25 ± 0.02a
Maximum UT in winter °C	23.21 ± 0.67	25.61 ± 0.77	27.61 ± 1.29	23.41 ± 1.78	24.34 ± 1.29	25.33 ± 0.89
Minimum UT in winter °C	8.37 ± 0.30	8.15 ± 0.40	8.59 ± 0.19	7.68 ± 0.53	7.93 ± 0.40	8.57 ± 0.15
Average UT in winter °C	13.47 ± 0.08 cd	13.76 ± 0.05 bc	14.26 ± 0.07 a	13.22 ± 0.06 d	13.54 ± 0.09 bc	13.78 ± 0.05 b
Maximum UM in spring %	30.09 ± 4.05	20.08 ± 1.81	21.41 ± 1.53	23.71 ± 4.61	17.40 ± 0.89	24.37 ± 5.26
Minimum UM in spring %	12.93 ± 2.16	12.18 ± 1.28	8.30 ± 0.70	11.73 ± 0.70	8.08 ± 0.38	8.14 ± 1.34
Average UM in spring %	15.67 ± 1.68a	14.95 ± 0.76ab	10.89 ± 0.76bc	13.98 ± 0.61ab	10.40 ± 0.26c	10.35 ± 1.06c
Maximum UM in summer and autumn %	23.86 ± 0.38ab	22.77 ± 1.49ab	19.93 ± 1.92b	22.91 ± 0.84ab	28.89 ± 3.68a	18.49 ± 1.54b
Minimum UM in summer and autumn %	16.08 ± 0.83a	13.79 ± 0.68ab	8.62 ± 0.58cd	12.56 ± 0.90abc	10.33 ± 1.65bc	4.56 ± 0.61d
Average UM in summer and autumn %	18.96 ± 0.68a	16.94 ± 0.48a	12.10 ± 0.46c	15.96 ± 0.74ab	13.61 ± 1.24bc	8.37 ± 0.42d
Maximum UM in winter %	8.61 ± 1.17	9.85 ± 1.12	13.23 ± 1.94	15.83 ± 0.91	13.39 ± 1.17	12.97 ± 1.43
Minimum UM in winter %	6.03 ± 1.19	7.64 ± 0.94	10.28 ± 2.08	13.24 ± 0.97	10.83 ± 0.95	11.10 ± 1.62
Average UM in winter %	7.33 ± 1.20	9.10 ± 1.08	11.50 ± 1.99	14.50 ± 0.98	11.97 ± 1.11	12.04 ± 1.47
Organic matter g kg^−1^	9.04 ± 1.20a	5.71 ± 0.78b	1.89 ± 0.15c	1.42 ± 0.11c	1.55 ± 0.09c	1.91 ± 0.14c
Total N g kg^−1^	0.58 ± 0.06a	0.43 ± 0.03a	0.21 ± 0.02c	0.27 ± 0.01c	0.30 ± 0.01b	0.31 ± 0.02b
Available N mg kg^−1^	55.47 ± 6.25a	36.77 ± 3.61b	24.21 ± 1.73c	29.11 ± 2.11 bc	30.76 ± 1.89 bc	30.60 ± 1.30 bc
Total P g kg^−1^	0.09 ± 0.00	0.09 ± 0.00	0.13 ± 0.04	0.08 ± 0.01	0.08 ± 0.01	0.09 ± 0.00
Available P mg kg^−1^	0.09 ± 0.03	0.07 ± 0.01	0.04 ± 0.01	0.06 ± 0.01	0.07 ± 0.01	0.05 ± 0.01
Total K g kg^−1^	1.98 ± 0.16	2.15 ± 0.20	2.15 ± 0.13	1.92 ± 0.32	1.63 ± 0.27	1.85 ± 0.23
Available K mg kg^−1^	18.11 ± 2.23a	12.62 ± 3.26ab	5.28 ± 1.46c	4.18 ± 1.11c	5.65 ± 1.44bc	3.07 ± 0.00c
pH	4.41 ± 0.03b	4.44 ± 0.03b	4.61 ± 0.02a	4.64 ± 0.03a	4.62 ± 0.03a	4.62 ± 0.02a

Different letters in the same column indicate significant differences at *P*<0.05.

### Main environmental factors affecting the distribution of *D*. *dichotoma*

**Fig 4:**
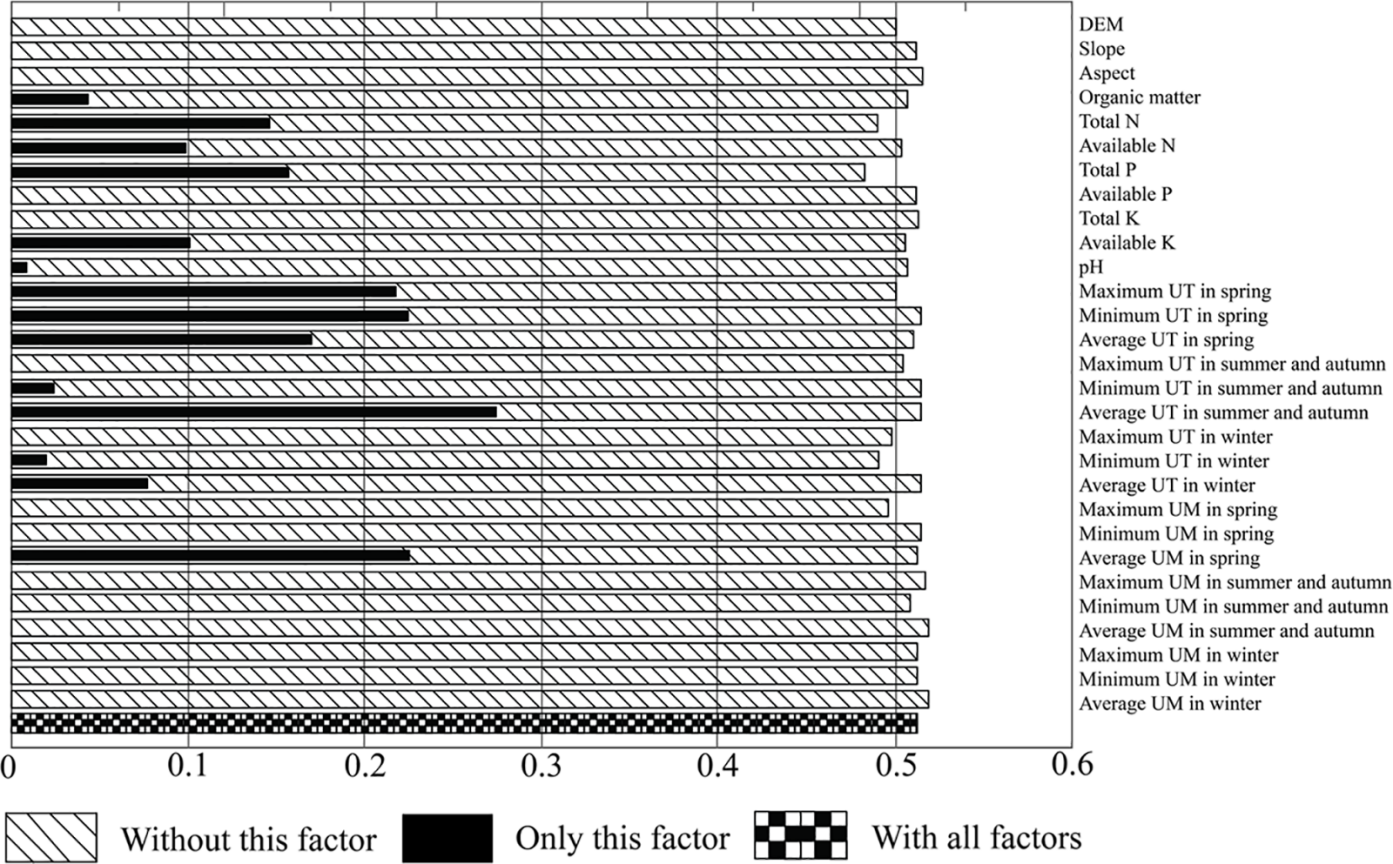
Environmental factors affecting the distribution of *D*. *dichotoma* in Laiyoukeng.

Five environmental factors largely contributed to the distribution of *D*. *dichotoma*: average UT in the summer and autumn, average UM in the spring, minimum UT in the spring, maximum UT in the spring, and average UT in the spring, followed by total N and total P; microtopography factors, on the other hand, had only marginal effects on the 29 factors in Laiyoukeng (Fig 4).

Average UT in summer and autumn, average UM in spring, minimum UT in spring, maximum UT in spring, average UT in spring, and total N showed significant differences among the three microtopographies (ridge, slope, and valley) in the gullies with or without *D*. *dichotoma* in Laiyoukeng (*P* < 0.05). Average UT in summer and autumn, maximum UT in spring, and average UT in spring tended to increase in the order from the valley through the slope to the ridge, while minimum UT in spring and average UM in spring showed the opposite trends. Total N also showed decreasing trends in the gullies with or without *D*. *dichotoma*. Total P was not significantly different among the three microtopographies (ridge, slope, and valley) in the gullies with or without *D*. *dichotoma* (Table 4).

### The potential distribution of *D*. *dichotoma*

**Fig 5:**
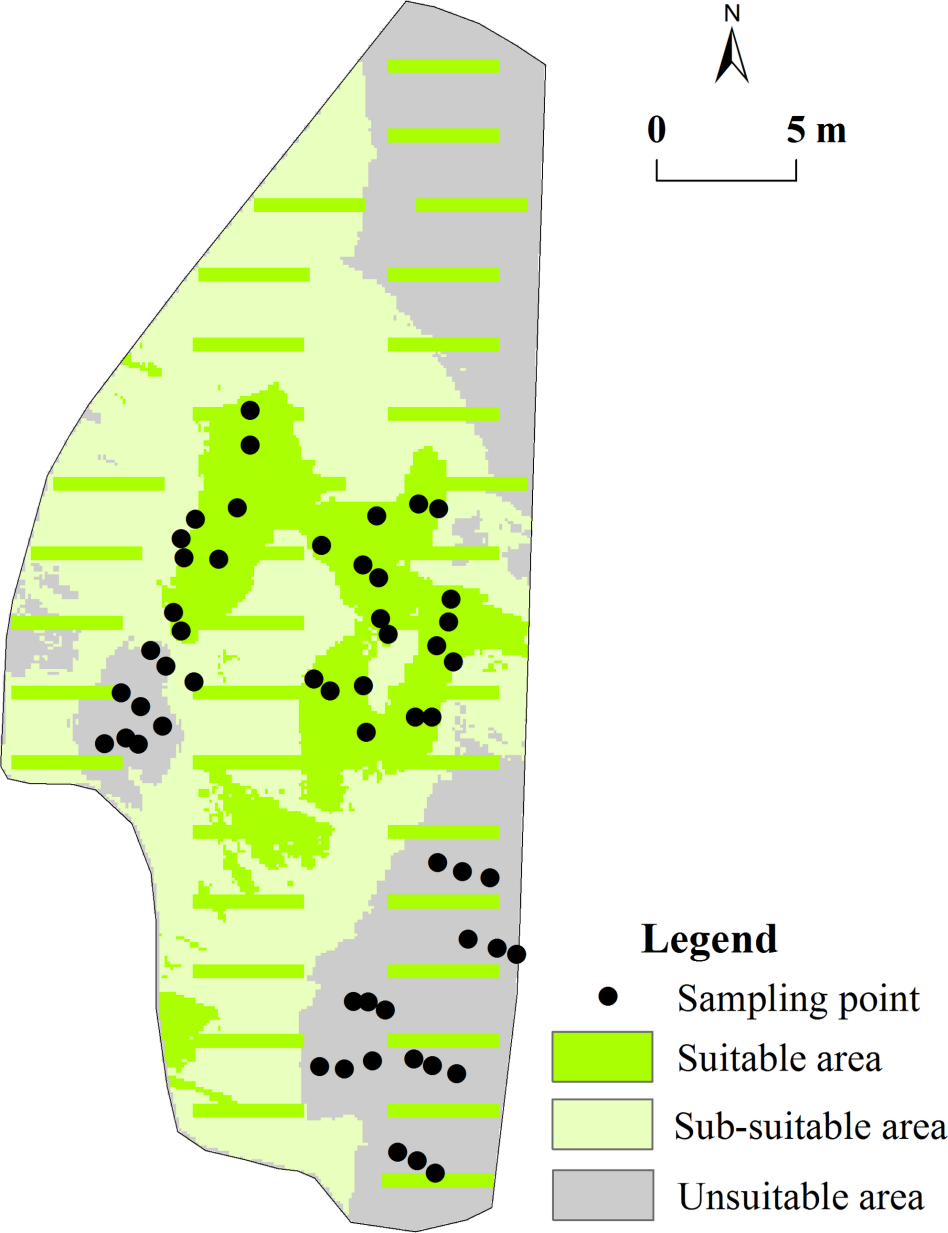
Potential distribution of *D*. *dichotoma* under the ABHMP scenario in Laiyoukeng.

The best selected model was the Generalized Linear Model with AUC 0.906. The predicted potential distribution of *D*. *dichotoma* using the Generalized Linear Model was 402.72 m^2^ (68.99%), and the unsuitable area was 181.02 m^2^ (31.01%) for *D*. *dichotoma* under the ABHMP scenario in Laiyoukeng. Out of a total of 402.72 m^2^, 152.36 m^2^ (26.10%) was identified as the suitable area and 250.36 m^2^ (42.89%) as the sub-suitable area for *D*. *dichotoma*. The suitable area was located mostly in the level trenches and valleys (Fig 5).

VC was 79.83%, 88.08%, 84.67%, and 67.33% in Duimountian, Longjing, Youfang, and Bashilihe, respectively.

## Discussion

### Microtopography had strong effects on the distribution and growth of *D*. *dichotoma*

In our study, we found that the percentages of microtopographies decreased in the order from the valley through the slope to the ridge in the *D*. *dichotoma* patches, most of the *D*. *dichotoma* physiological factors decreased in the order from the valley through the slope to the ridge, and the valley provided gentler temperatures and higher humidity than the ridge. This indicates that microtopography has strong effects on the distribution and growth of *D*. *dichotoma*, and the valley is more suitable for *D*. *dichotoma*. With regard to soil and water, the valley is a run-on area, in the slope run-on equals runoff, and the ridge is a runoff area [32]. Soil and water loss in a runoff area results in poorer water, less soil nutrient content, and lower soil depth and stability, with subsequent negative effects on plants such as seed death [33]. A run-on area, where runoff soil and water converge, tends to have better plant growth and development [34]. For example, the soil seed bank and ground vegetation decreased in the order of bottom slope > lower side slope > middle side slope > upper side slope > crest slope in fixed sand of the Mu Us sandy land, China [35]; and in the hilly Loess Plateau region of China, the biomass of the grassland community and annual fine root production were in the order of lower > middle > upper > top in the shady slope [36]. Thus, the valley usually becomes the microrefugium, a small area with local favorable environmental features in which small populations can survive outside their main distribution area, protected from unfavorable regional environmental conditions. For example, relict glacial bodies and active geomorphological processes along alpine valleys favor microrefugial niches where alpine species are able to survive in Alpine regions during interglacial phases [18].

### Main environmental factors affecting the distribution of *D*. *dichotoma*

The microclimate factors were the most critical environmental factors affecting the distribution of *D*. *dichotoma*, followed by the soil factors, while the microtopography factors only had marginal effects. Temperature-related factors in summer and autumn, and spring, and moisture-related factors in spring, were the most important microclimate factors. In previous reports, temperature and moisture have been identified as major factors influencing *D*. *dichotoma* in terms of growth, survival, metabolic rate, reproduction, and dispersal [37]. If a soil is hot and dry, the root and rhizome of *D*. *dichotoma* cannot survive, especially when the soil temperature is over 45°C [38]. It has been reported that *D*. *dichotoma* starts to sprout and its roots, rhizomes, and leaves start to grow in spring [39]. Therefore, temperature and moisture levels in spring are important drivers shaping the distribution of *D*. *dichotoma*.

One of our previous studies showed that total P was low due to severe soil and water loss in the red soil hilly region of China [24]. In another of our studies, the coverage, total biomass, and height of *D*. *dichotoma* were positively correlated with total N [22]. This suggests that the distribution of *D*. *dichotoma* may be constrained by total N and total P. However, in the present study, total P was not significantly different among the three microtopographies in the gullies with *D*. *dichotoma* and gullies without *D*. *dichotoma*, with the opposite trend being observed for total N in the gullies with or without *D*. *dichotoma*. This result was unexpected and difficult to explain, and the effects of soil on *D*. *dichotoma* and the effects of soil on *D*. *dichotoma* need to be investigated further.

Interestingly, the microtopography factors had only marginal effects on the distribution of *D*. *dichotoma*, which seemed contrary to the distribution and growth of *D*. *dichotoma* among microtopographies. We used both the TPI and the slope to form 6 microtopographies including valley, lower slope, flat slope, middle slope, upper slope, and ridge. The microtopography in this study is an integration of microtopography factors including topographic positions, altitude, and slope which can act either indirectly, by modulating microclimatic and soil properties, or directly, through slope and associated gravitational processes [40]. Altitude, slope, and aspect are likely the same in different microtopographies; for example, slope and aspect are very similar in valleys and ridges with different altitudes while altitude, slope, and aspect may be different within the same microtopography; for example, aspects are opposite in two upper slopes. Consequently, microtopography is a comprehensive variable and altitude, slope, and aspect cannot completely replace microtopography. It has been argued that topographical factors are not important for plant distribution [41]; for instance, although altitude was not specifically selected, climate was shown to be a better and more detailed predictor of red panda conservation than altitude *per se* [42]. Further research is needed to find out more appropriate microtopography factors to represent microtopography and better understand how microtopography affects other environmental factors and plant distribution.

### Effects of ABHMP on the potential distribution of *D*. *dichotoma* and implications for ecological restoration

The predicted potential distribution of *D*. *dichotoma* under the ABHMP scenario was nearly three times higher than the current distribution, and the suitable area was located mostly in the level trenches and valleys in Laiyoukeng. The VC in Laiyoukeng was in the range of Duimountian, Longjing, Youfang, and Bashilihe, which indicated that the prediction was accurate to some extent. The results showed strong effects of ABHMP on the potential distribution of *D*. *dichotoma*, and SDMs proved to be a valuable tool to identify the main environmental factors, predict potential species distribution, and assess ecological restoration measures at the microscale. Our method can provide a geographic template that can potentially be applied to a diverse set of areas and species. The main requirement for using the template is the availability of good quality datasets, but acquiring datasets is difficult. Recently, remote sensing has achieved the high spatial resolution and physical accuracy needed to model species distribution [43]. For example, hyperspectral sensors mounted on satellites or airplanes can now gather data enabling the calculation of temperature or moisture variables at a high resolution, in addition to raw bands or vegetation indices [40]. Determining the presence and absence of species in the good quality datasets mentioned above generally requires a considerable amount of fieldwork, which then decides what kind of SDMs should be used. SDMs can be roughly divided into two groups, depending on the origin of the species: presence–absence and presence-only SDMs [44]. Presence–absence SDMs use information about locations where the species is found and not found, whereas presence-only SDMs usually search for correlations between environmental parameters and observation records [45]. Because datasets with recorded absences of species are scarce, background or pseudo-absence locations are widely used [44], leading to sampling bias [46]. Presence–absence SDMs may result in higher accuracy when reliable presence–absence information is available [45]. In this work, we indicated locations where *D*. *dichotoma* was absent with high precision using continuous observation from 2012 to 2016. In addition, the distribution pattern of species and environment factors is an important aspect of the good quality datasets, which has major influence on interpolation methods. The 54 sampling points used to create the soil factor layers and microclimate factor layers are aggregated in space, which would lead to errors to some extent in some areas without points in our study. Thus, the effects of the distribution pattern of species and environment factors on SDMs are in need of further study.

## Conclusions

Microtopography had strong effects on the distribution and growth of *D*. *dichotoma*, and the valley was the most suitable for *D*. *dichotoma*. Microclimate factors were the most critical environmental factors for the distribution of *D*. *dichotoma*, followed by soil factors, whereas microtopography factors had only limited effects. Microtopography is a comprehensive variable and altitude, slope, and aspect cannot completely replace microtopography. The predicted potential distribution of *D*. *dichotoma* under the ABHMP scenario, using SDMs, was accurate. ABHMP had strong effects on the potential distribution of *D*. *dichotoma*, and SDMs proved to be a valuable tool for identifying the main environmental factors, predicting potential species distribution, and assessing ecological restoration measures at the microscale.

## Supporting information

S1 File Layers. (ZIP)

S2 File Soil and microclimate factor. (DOCX)

S1 Table Physiological factors, soil factors and microclimate factors. (XLS)
