# Supplementary figures and images for: Effects of ecological restoration measures on the distribution of Dicranopteris dichotoma at the microscale in the red soil hilly region of China
Source: PLoS One. 2018 Oct 24;13(10):e0204743. doi: 10.1371/journal.pone.0204743 (PMC6200197; doi:10.1371/journal.pone.0204743)

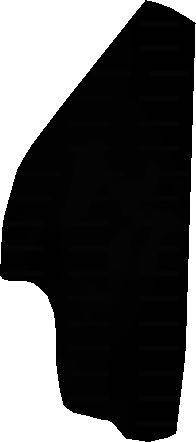

Supplement: S1 File — (ZIP) [file pone.0204743.s001.zip › Layers/Potential distribution of Dicranopteris dichotoma.tif]

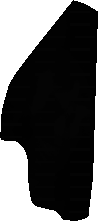

Supplement: S1 File — (ZIP) [file pone.0204743.s001.zip › Layers/Potential distribution of Dicranopteris dichotoma.tif.ovr]

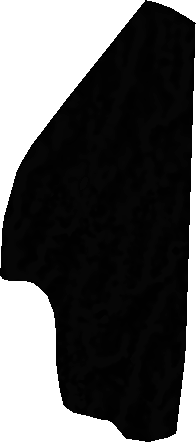

Supplement: S1 File — (ZIP) [file pone.0204743.s001.zip › Layers/microtopography.tif]

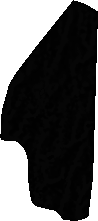

Supplement: S1 File — (ZIP) [file pone.0204743.s001.zip › Layers/microtopography.tif.ovr]

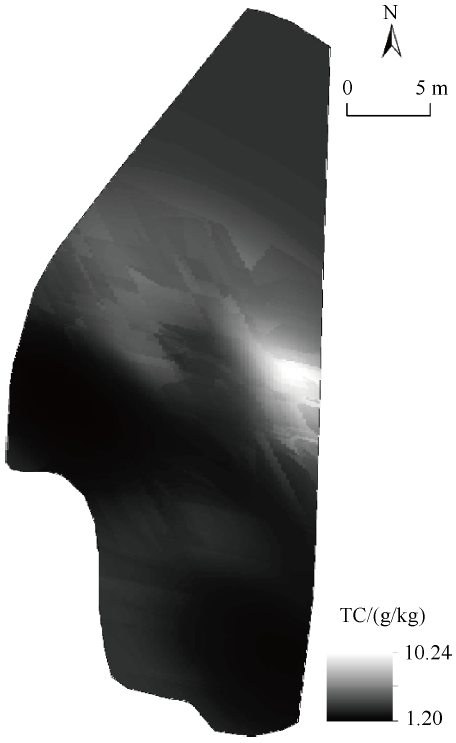

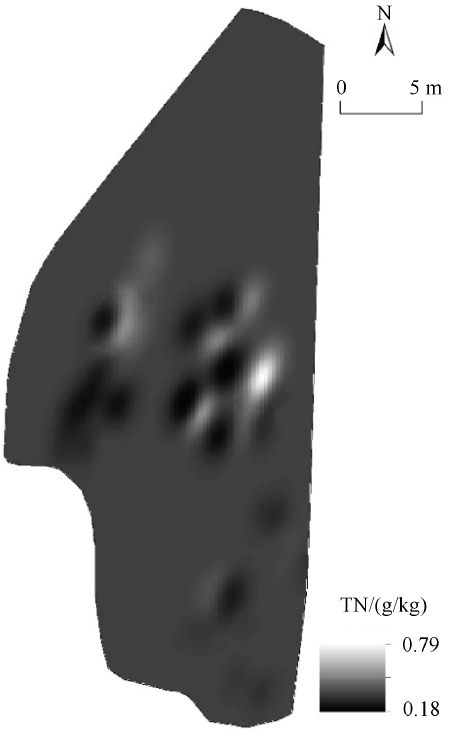

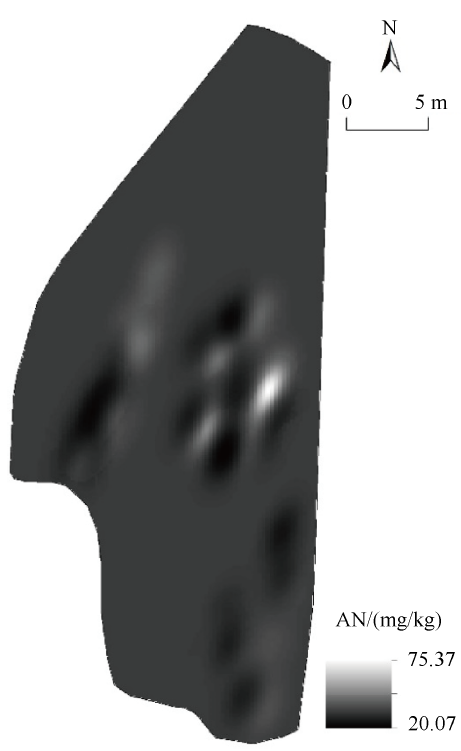

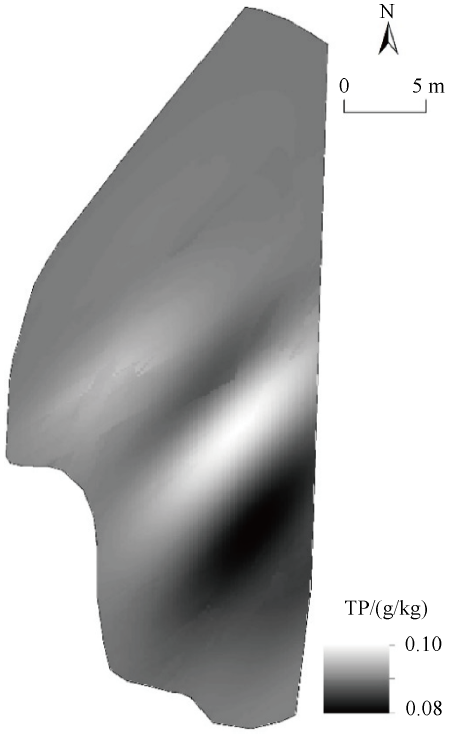

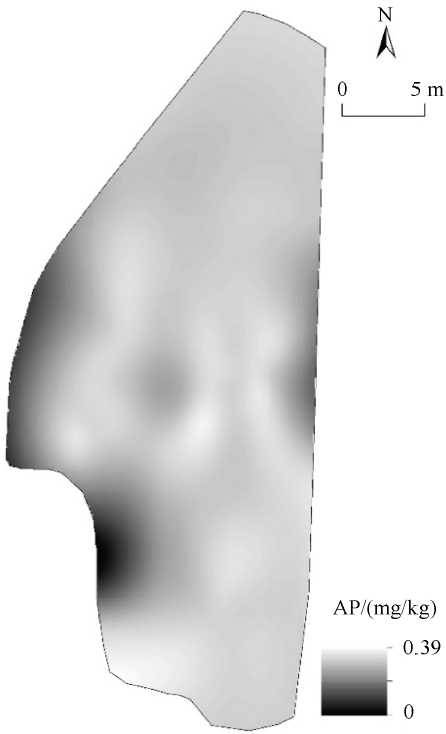

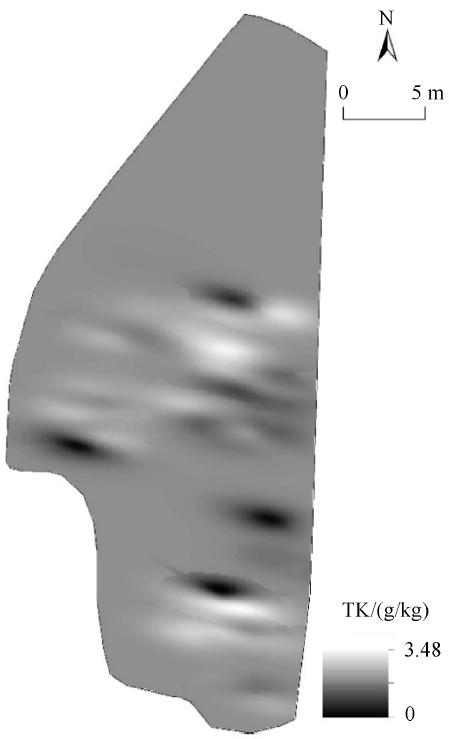

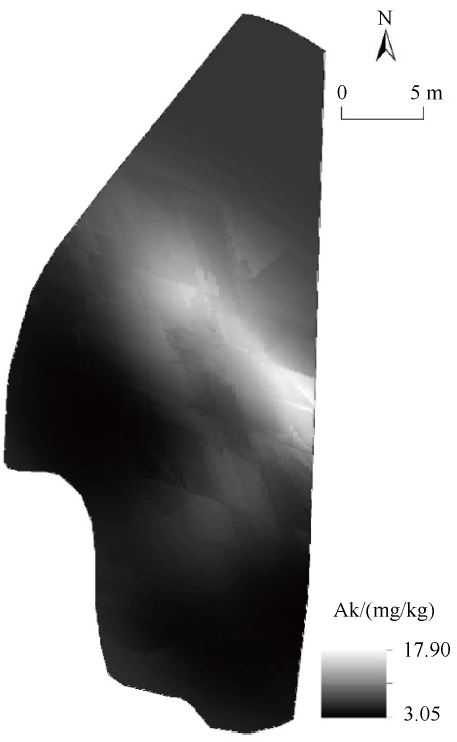

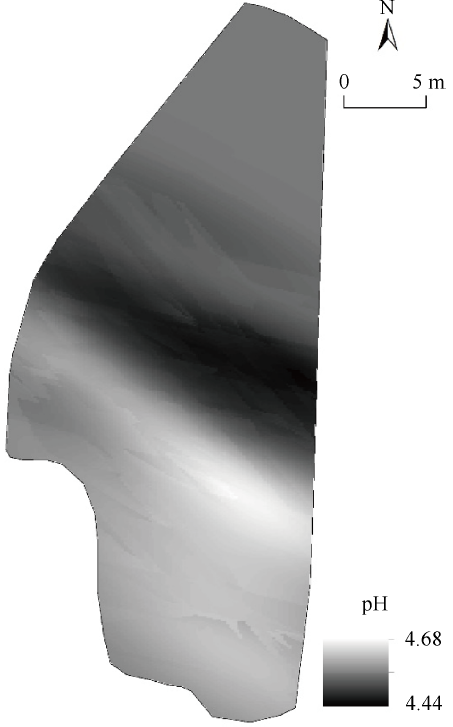


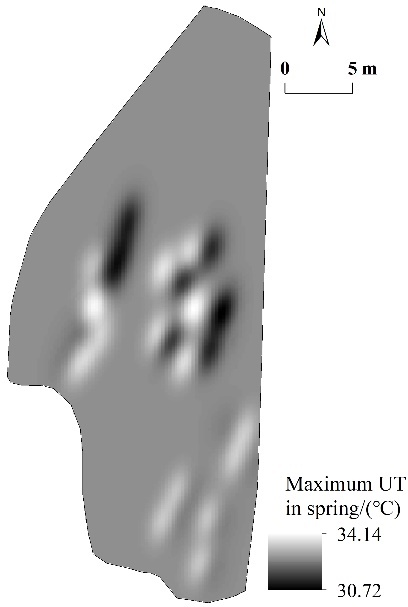

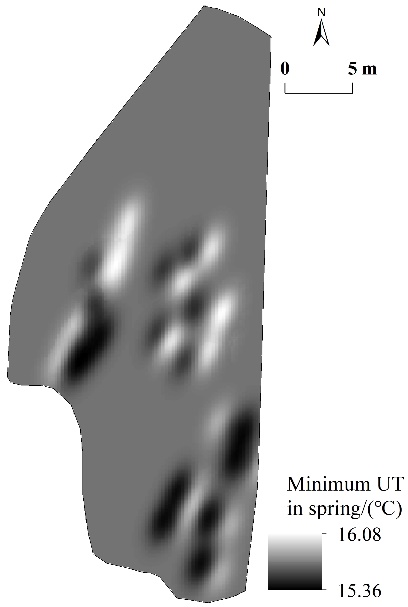

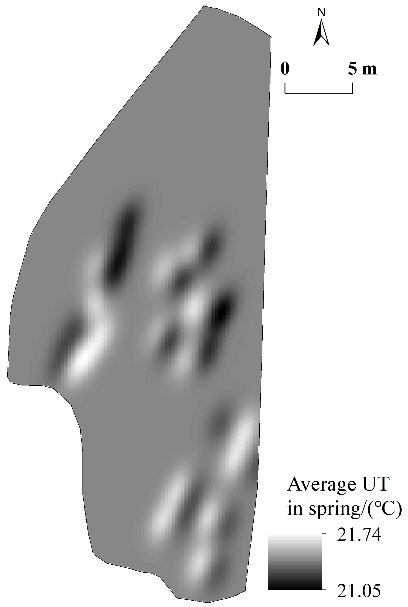


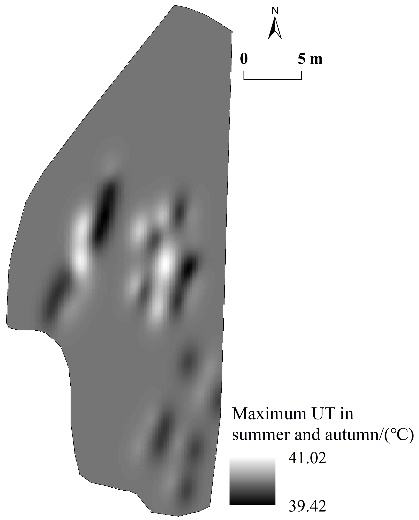

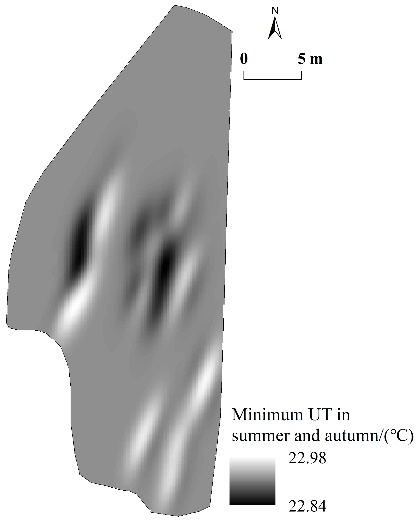

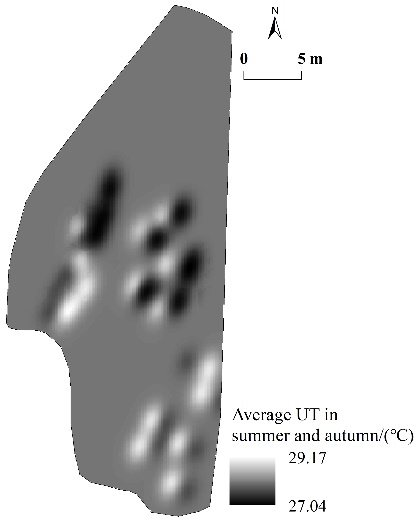


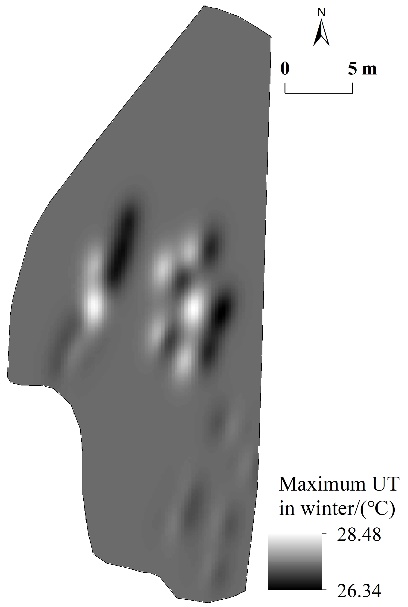

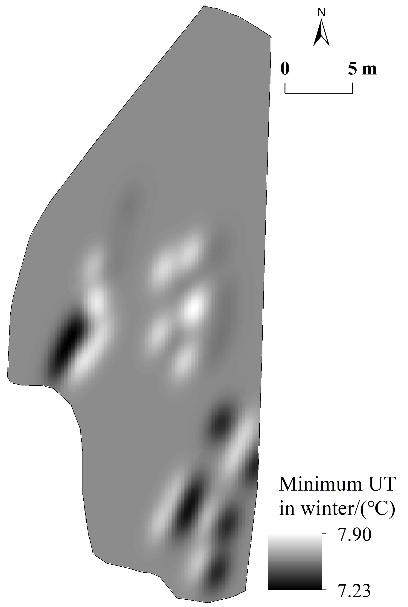

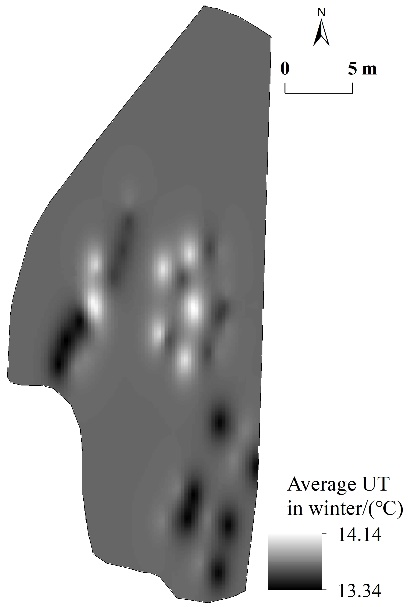


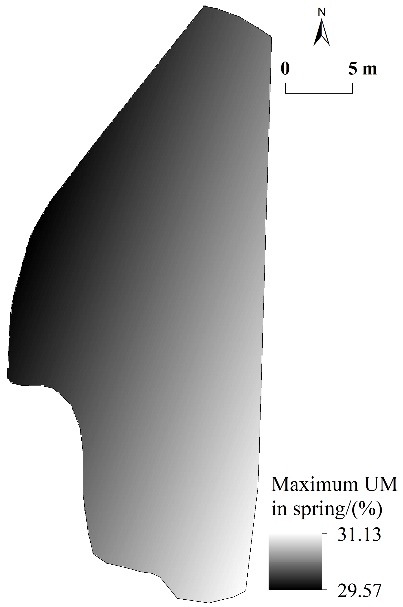

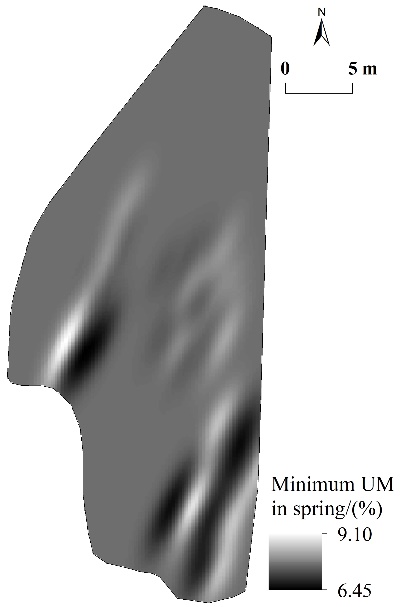

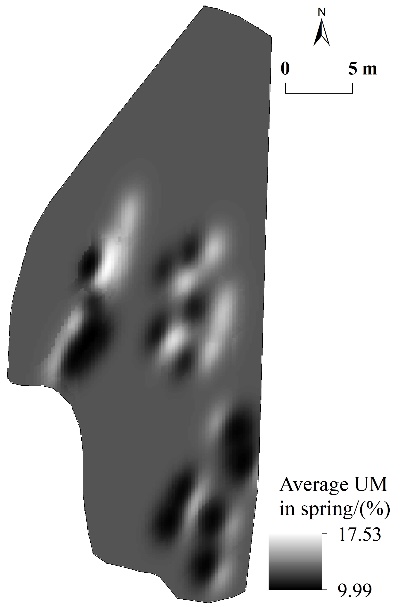


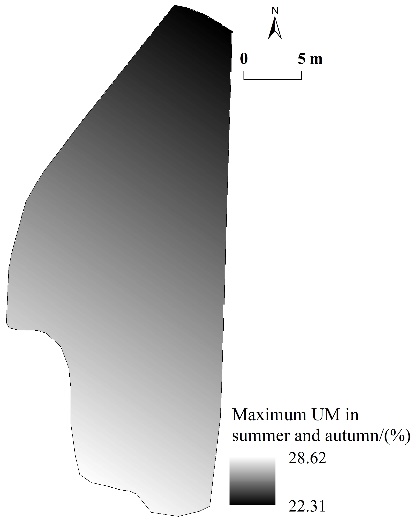

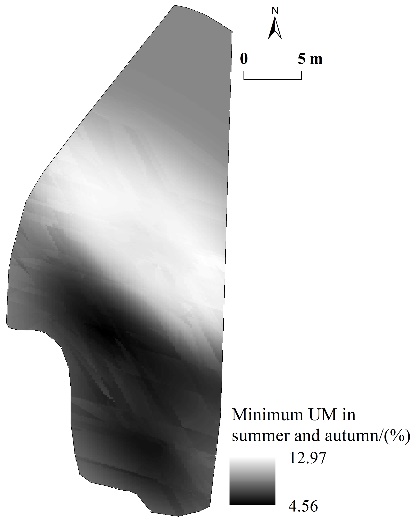

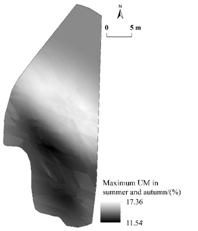


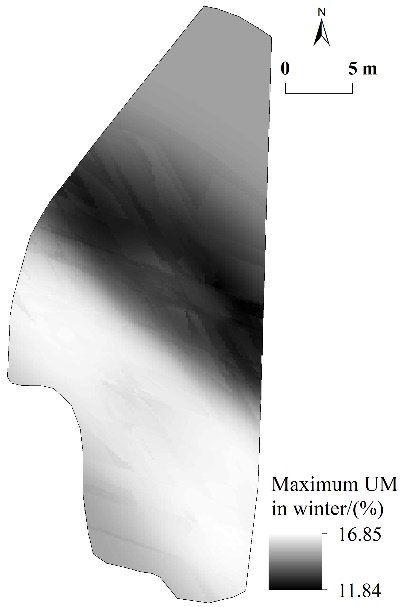

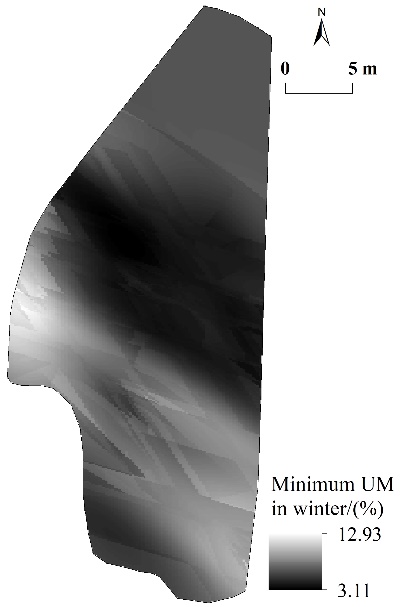

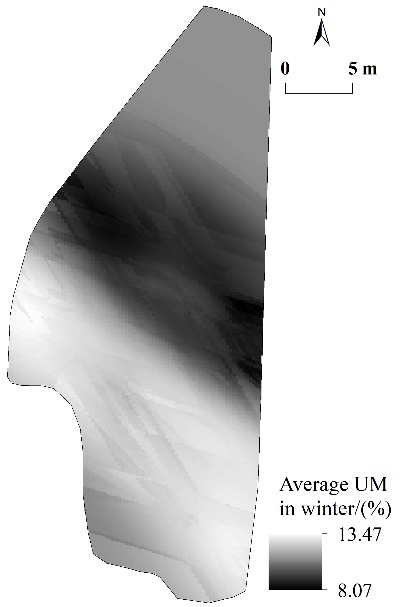

Supplement: S2 File — (DOCX) [file pone.0204743.s002.docx]
